# The Current and Novel Imaging Modalities for Ocular Vasculitis in Behcet’s Disease: A Review

**DOI:** 10.7759/cureus.69528

**Published:** 2024-09-16

**Authors:** Mandeep Kaur, Kevin Yip

**Affiliations:** 1 Ophthalmology, Government Medical College, Patiala, IND; 2 Division of Rheumatology, Department of Internal Medicine, Wyckoff Heights Medical Center, Brooklyn, USA

**Keywords:** autoinflammatory, behcet disease, fluorescein angiography, oct angiography, retinal vasculitis

## Abstract

Behcet's syndrome is a multisystem inflammatory disorder characterized by a chronic relapsing course and diverse clinical manifestations, prominently affecting young adults along the ancient Silk Road and beyond. The disease pathogenesis involves complex interactions between genetic predisposition, environmental triggers, and dysregulated immune responses, leading to systemic vasculitis and tissue damage. Ocular involvement, a hallmark of Behcet's Disease (BD), significantly impacts morbidity, with uveitis as a common initial presentation that can progress to severe vision-threatening complications like retinal vasculitis and occlusive disease. This review consolidates current knowledge on ocular manifestations in BD, emphasizing the pivotal role of multimodal imaging in diagnostic evaluation and management. Fundus photography serves as a baseline tool for documenting intraocular lesions and monitoring treatment responses. Fluorescein angiography remains the gold standard for detecting acute inflammatory changes and vascular leakage patterns essential for disease staging and prognostication. Recent advancements in imaging, such as ultra-wide field imaging (UWF), indocyanine green angiography (ICGA), Doppler ultrasonography, Optical Coherence Tomography (OCT), OCT angiography (OCTA), adaptive optics (AO), and retinal function imaging (RFI), provide unprecedented insights into microvascular dynamics, structural changes, and functional impairments associated with ocular BD. Integration of these advanced imaging modalities enhances early detection of subclinical disease, facilitates precise localization of inflammatory lesions, guides therapeutic interventions, and monitors treatment efficacy. OCT and OCTA, in particular, offer non-invasive, high-resolution assessments of macular edema, vascular perfusion abnormalities, and choroidal thickness alterations critical for optimizing patient care. In conclusion, multimodal imaging represents the cornerstone in the comprehensive management of ocular manifestations in Behcet's Disease, offering clinicians invaluable tools for accurate diagnosis, treatment planning, and long-term monitoring of disease progression and treatment outcomes.

## Introduction and background

Behcet's syndrome is primarily a systemic vasculitis affecting arteries and vessels of all calibers [1], characterized by a chronic relapsing and remitting inflammatory course with multi-system involvement. Historically, Behcet's disease (BD) cases were clustered along the ancient Silk Road, with a high prevalence in Turkey [2]. However, there are currently wide variations based on ethnicity and location [1], affecting individuals aged 15-45 years [3], with young males showcasing severe disease and worse prognosis [4]. Similar to other autoimmune diseases, the pathophysiology of Behcet's disease involves exposure to numerous environmental triggers in genetically predisposed individuals that activate an inflammatory cascade, precipitating an autoinflammatory process. Pro-inflammatory cytokines, including TNF-alpha, interferon-gamma, IL-17, IL-23, and cytotoxic T cells, are induced by T helper cells fostering a neutrophil response, promoting a procoagulant state [5]. The HLA-B51 allele plays an essential part in prevalent areas by not only affecting incidence, which varies from 55% among carriers compared to 10-15% in non-carriers, but also affects severity due to its association with posterior uveitis and neuro-Behcet [6]. Behcet's virtually affects all body systems, including but not limited to non-scarring oral ulceration being the initial symptom, scarring genital ulceration, erythema nodosum commonly in females, pseudofolliculitis in males, superficial migratory thrombophlebitis more commonly in males [5], mono or polyarticular nondestructive arthritis, more commonly the knee joint, gastrointestinal mucosal ulceration commonly involving the ileocecal region which could be hard to differentiate from inflammatory bowel disease [7]. Neurological involvement is usually chronic and progressive, with 10% of cases having retrobulbar neuritis, disc edema, oculomotor and abducens nerve palsy, meningoencephalitis, myelitis, and dementia in 30% of cases. Systemic vasculitis causes superficial and deep venous thrombosis and aneurysms affecting cerebral, pulmonary, and cardiac vasculature [5,7].

Ocular Behcet disease is a riveting pathology and part of a multi-system syndrome. Ten percent of cases may present with ocular manifestations at an average age of the early 20s, with uveitis being the presenting clinical entity with an incidence of 6-20%. Isolated vitritis is a common feature in early ocular BD. The disease spectrum varies from inflammation of the episclera, sclera, cornea, anterior chamber, uvea, retina, ocular vasculature, orbit, optic nerve, and extraocular muscles. Ocular inflammation is similar and, simultaneously, different in many aspects compared to systemic disease. Inflammatory findings could be self-limiting, but recurrent disease increases ocular morbidity, and retinal vasculitis is at the forefront for a worse visual prognosis [4]. Like systemic disease, the HLA-B51 allele is associated with a severe form of ocular BD in 51% of cases compared to 31% in non-carriers [6]. Posterior uveitis can align with parenchymal involvement in neuro-Behcet's disease [5]. Retinal vasculitis is distinct from systemic vasculitis, the only vascular inflammation that can be diagnosed, monitored, and prognosticated with direct visualization. BD affects both arteries and veins regardless of vascular caliber, with segmental or confluent involvement. Pathologically, perivascular lymphoplasmacytic infiltration is seen [8]. Retinal vasculitis in BD causes active inflammation, which is identified with perivascular exudates, retinal swelling, macular edema, and chronic sequelae of occlusive disease. Central vein occlusion and arteriolar occlusions are less common, around five percent, compared to branch vein occlusions, which can be seen in 33% percent of cases of ocular BD and are notorious for causing macular edema and visual impairment. The occlusive disease causes ischemia in around 1% of cases, leading to the development of new vessel formations that are friable compared to normal vessels, causing leakage and retinal edema [9].

Multimodal imaging integrated with clinical evaluation benchmarks the diagnosis of retinal vasculitis. Identifying microcirculatory changes in retinal vasculature requires expertise. Advancements in new imaging techniques can help with early detection, delineation of etiology, treatment, and monitoring of disease activity that would help decrease ocular morbidity. This review focuses on consolidating available imaging techniques and outlining how clinicians can use these to assist in diagnosing and managing BD [10].

## Review

Fundus photography

Fundus photography is the keystone for the visualization of intraocular lesions. It helps document the lesions, compare them in follow-up visits, and monitor lesions during treatment. Ocular BD commonly has a waxing and waning course [11]; documenting ocular lesions is very important as many other diseases causing posterior uveitis behave differently, and fundus photography can also aid in differential diagnosis. A combination of vitreous haze, retinal infiltrates, and occlusive retinal vasculitis can be identified on fundus examination. The extent of ocular inflammation can also be quantified based on vitreous haze. With anti-inflammatory therapy, a vitreous haze can be seen in resolution after weeks of treatment, and it behaves differently from intermediate uveitis [12].

Similarly, retinal infiltrates are also transient compared to other chorioretinitis. Moreover, old sequelae from previous episodes can also be documented, like the retinal nerve fiber layer (RNFL), which can be detected with fundus photography. Retinal infiltrates are foci of retinal inflammation, which are superficial and distinctively in BD. Unlike other inflammatory pathologies, the infiltrates do not obscure underlying vessels [12]. Retinal peri-vascular inflammation is a distinctive feature of ocular BD, with more frequent venous involvement than arterial. Retinal vein occlusions and periphlebitis on fundus examination can be seen as vascular sheathing, diffuse perivenous haziness, and hemorrhages [11]. Though fundus photography is a preliminary clinical tool, further diagnosing the extent of vasculitis and further guiding the management of fundus angiography is essential.

Fundus fluorescein angiography

Fluorescein angiography (FFA) is a diagnostic method that involves capturing a sequence of images after the intravenous administration of sodium fluorescein. Accurate interpretation and comprehension of FFA are essential for diagnosing and evaluating numerous ocular vascular conditions [13]. FFA has been used since 1962 but remains the gold standard for detecting acute inflammatory lesions and occlusive disease in inflammatory retinal disorders. Preclinical ocular BD can be identified with optic disc staining and peripheral capillary leakage, even without distinctive clinical disease [14]. Certain features, such as increased vascular tortuosity of the retinal veins, staining of the optic disc and vessel wall, and leakage from retinal vessels, indicate the acute inflammatory phase of ocular vasculitis. Diffuse vascular leakage in a fern-like pattern is more frequently seen in BD and is also seen in chronic phases of inflammation [15]. FFA can also help diagnose occlusive retinal disease; inflammation can lead to vessel occlusion, leading to non-perfusion, promoting angiogenesis and neovascularization (NV) [16]. Identification of these lesions is essential as it would help direct treatment. Many occlusive lesions need photocoagulation; however, optic disc NV behaves differently in ocular BD. FFA can help differentiate between occlusive versus capillary leakage, leading to optic disc NV [17]. Besides diagnostic value, FFA can help showcase some poor prognostic factors for ocular BD, like optic disc NV and macular ischemia [18]. Kim et al. studied the correlation between FFA findings and visual acuity (VA). It was observed retrospectively that mean VA in patients with posterior pole vasculitis, like macular leakage and diffuse capillary leakage, was statistically significantly worse than peripheral retinal involvement [19]. Keorochana et al. studied the role of FFA in prognosticating visual outcomes in ocular BD. Behçet's disease ocular attack score of 24 is a 24-point based on the location of lesions, scoring is based on the extent of inflammation in different parts of the eye, namely anterior chamber, vitreous, peripheral retina, posterior pole, fovea and optic disc. These scores can also be used to compare post-treatment outcomes and resolution of lesions. A study done by Keorochana et al. [20] depicted a point-based scoring to evaluate the degree of inflammation in ocular tissue involving the anterior chamber, posterior chamber, optic disc, and central and peripheral retina. Though a small study, they showed that BOS24 scores ≥ 6 are statistically significantly associated with worse visual outcomes [20].

Ultra-wide field imaging

A routine fundus camera captures only 30° to 60° of the fundus at a time, and the entire retina cannot be imaged in one click, so peripheral lesions can be easily missed. Ultra-wide field imaging (UWF) can provide imaging of up to 200 degrees of retina [21]. UWF makes detecting peripheral leakage, NV, and ischemia in ocular BD easier. UWF can significantly enhance diagnosing, monitoring, and treating retinal vasculitis in Behçet's disease patients. As quoted earlier, preclinical lesions are usually peripheral; UWF can prevent missing these lesions. Studies have shown that UWF detected 10% more disease activity than FFA and changed management in 63% of cases [22].

**Figure 1:**
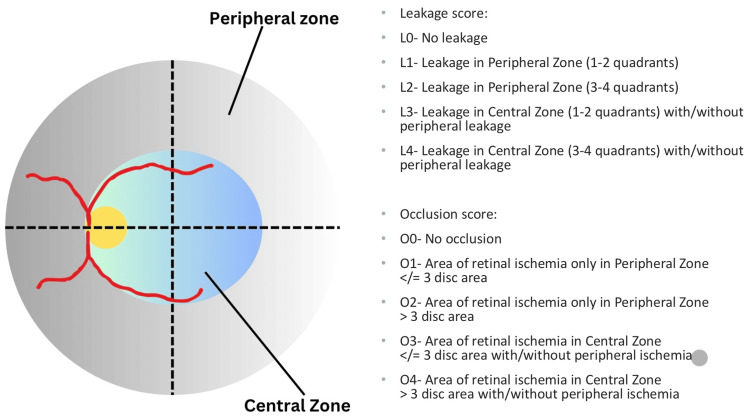
Retinal vasculitis grading Figure 1 shows a fundus image, with retinal vessels, the central zone is defined as part of the retina in the circle formed by the fovea as the center and distance between the fovea and the nasal edge of the optic disc as the radius and the rest of retina outside central zone is defined as peripheral zone [23]. (Source is open access)

Dhirachaikulpanich et al. published a study demonstrating a new grading scheme to assess both the leakage and occlusive phase of vasculitis using UWF; the central zone is defined as a circle made with the fovea as the center and distance between the fovea and the nasal edge of the optic disc as the radius and peripheral zone are defined as area peripheral to the central zone. There are no standard scoring systems to quantify retinal vasculitis; Figure 1 is adapted from a study by Dhirachaikulpanich et al. that can help prognosticate retinal vasculitis. Higher scores were associated with worse visual activity at follow-up visits, too. This scoring was also tested for inter- and intra-observer reliability, and good agreement was shown among observers [23].

Indocyanine green angiography

Indocyanine green angiography (ICGA) helps to visualize choroidal circulation and is preferred over FFA for choroidal pathologies as retinal pigment epithelium obscures choroid [24]. BD is systemic vasculitis, and it affects choroidal vessels along with retinal vessels. There are no distinctive features individualized in BD if choroid is involved. It may show filling defects, staining of vessel walls, and hypofluorescent patches. As hyperfluorescence slowly increases on ICGA, extensive choroidal vessel leakage can be seen in BD. ICGA may not present with distinctive characteristics in BD but can help diagnose the duration of ocular disease. The hypofluorescent lesions that become isofluorescent in ICGA are seen in diseases with shorter duration; however, if hypofluorescent lesions remain hypofluorescent, even in the late phases of BD, they are usually seen in chronic ocular BD. ICGA and FFA can both be used complementary to each other [25]. ICGA can also aid in differentiating ocular BD from other primary choroidal inflammatory diseases, which will determine the course of treatment.

Doppler ultrasonography

Duplex vessels and color Doppler sonography can detect Doppler shifts, approximate flow velocity, and vessel course, even in small retinal vessels. Doppler sonography has been used extensively to study orbital pathologies. Ozdemir et al. studied the use of color Doppler for ocular involvement [26]. In ocular BD, it was observed that there was a reduction in peak systolic and diastolic velocities of retinal vessels. BD leads to severe impairment in the hemodynamics of the central retinal artery and posterior ciliary vessels due to increased vascular resistance. Sonography is simple, noninvasive testing that can help screen patients for ocular BD, as decreased flow in the central retinal artery could point towards early ocular involvement [27]. Blood flow velocities in the central retinal artery are significantly lower in severe disease than in mild or moderate disease. Sonography can help document significant circulatory changes in retinal vasculature in BD [28].

Optical coherence tomography

Optical coherence tomography (OCT) uses low-coherence interferometry and consecutive beams to recreate cross-sectional slices by combining A-scans and B-scans. Initially, OCT utilized time-domain technology, which has since been replaced by spectral-domain OCT (SD-OCT), dramatically increasing imaging quality with higher resolution [29]. SD-OCT is instrumental in diagnosing, monitoring disease activity, and assessing the prognosis of Behçet's Disease (BD) [30]. Macular edema, a common complication of retinal vascular and inflammatory diseases [31], can be determined using OCT. This imaging technique aids in screening for and monitoring disease activity [32]. OCT can delineate the extent and placement of fluid within retinal layers, distinguishing between diffuse, subretinal, cystic, and serous detachments, and can also help visualize vitreoretinal interface abnormalities. As a noninvasive technique, OCT can differentiate acute from chronic BD.

Acute BD usually presents with exudative macular detachment, whereas cystoid macular edema is seen in chronic disease [33]. Research has shown that central macular thickness and macular volume are significantly increased in patients with posterior segment involvement compared to those without, suggesting a breakdown of the blood-retinal barrier. These anatomical changes tend to decrease after successful treatment. Thus, OCT is a vital tool for assessing macular complications in BD [30]. OCT can detect foveal thickness and disruptions in the ellipsoid zone, which are related to worse visual outcomes. Macular thinning is associated with the chronicity of the disease, while leaky vasculature points towards an acute inflammatory phase [34]. The transient nature of retinal infiltrates in ocular BD is also evident in OCT.

Additionally, OCT can detect associated retinal nerve fiber layer defects in posterior pole disease in BD [16]. OCT also measures choroidal thickness using enhanced depth imaging settings. Several small-scale studies have shown that subfoveal choroidal thickness is related to the severity of inflammation. The choroid was thicker in BD patients than in normal eyes, though most findings were statistically insignificant [35,36].

Optical coherence tomography angiography

Advanced techniques incorporated into OCT can help deduce vascular hemodynamics and perform detailed anatomical studies of the retinal vasculature. While fluorescein angiography (FFA) remains an invasive procedure, OCT angiography (OCTA) has emerged as a noninvasive alternative, beneficial for diagnosing microvascular changes in BD. OCTA can delineate the superficial and deep capillary plexuses (SCP and DCP), which are distinctively involved in ocular BD. This technique allows for measuring the foveal avascular zone (FAZ), which is more prominent in patients with ocular BD than in healthy subjects. Additionally, eyes with BD have shown peripapillary capillary disruption, which OCTA can detect with high precision [37,38].

OCTA can objectively identify lesions not visualized on FFA, with peripapillary retinal thickness increasing without FFA changes [39]. This capability makes OCTA particularly valuable for preclinical and clinical diagnosis and for monitoring disease activity and determining prognosis in BD patients. By comparing SCP before and after treatment, clinicians can gain insights into the efficacy of therapeutic interventions. However, it should be noted that DCP and FAZ are considered poor prognostic indicators in BD [40]. Nonetheless, the ability of OCTA to provide detailed visualizations of the retinal microvasculature and track changes over time makes it an indispensable tool in the management of ocular BD. FFA can detect 52% of clinically inactive ocular BD, with vasculitis being seen in 30% of cases, while in active cases, FFA detected 45% of vasculitis cases. Though FFA is invasive, it is an essential tool for seeing capillary leakage, which is not evident on OCT [41]. OCT has been shown to detect 74% of subclinical microvascular and structural modifications in the retina and the choroid [42]. Subfoveal choroidal thickness and FFA leakage scores are notably high in the acute inflammatory phase compared to the recovery phase. Hence, these qualitative measurements may prove beneficial in determining treatment and maintenance therapy [43]. OCT and OCTA are excellent when we need interpretation of macular edema, and they are incomparably superior in evaluating changes in capillary plexuses with enhanced depth imaging [44].

Overall, OCTA complements the capabilities of traditional OCT, offering a comprehensive approach to understanding and managing retinal vascular conditions associated with Behçet's Disease. Its noninvasive nature and ability to provide detailed insights into microvascular changes make it a valuable addition to ophthalmologists' diagnostic and monitoring toolkit.

Adaptive optics

Adaptive optics (AO) represents a significant advancement in ophthalmic imaging, particularly in visualizing retinal photoreceptors and capillaries with unprecedented clarity and detail. This technology enables high-resolution imaging and quantitative analysis of these microstructures, providing valuable insights into their morphology and function. AO is exceptionally sensitive in detecting and measuring perivascular infiltration at the microscopic level, making it a promising tool for understanding the pathophysiology of various ocular diseases, including those involving inflammatory processes [45]. For instance, perivascular cuffing secondary to inflammation has been identified as a potential cause of vascular occlusions. AO can precisely measure the size and extent of these perivascular cuffs, correlating these findings with clinical outcomes. In the context of Behçet's Disease (BD), AO shows promise as a diagnostic tool specifically for assessing the peripapillary region rather than peripheral lesions [46]. This capability is crucial as peripapillary involvement can significantly impact visual function and prognosis in BD patients. Overall, AO enhances our ability to visualize and quantify subtle changes in retinal microstructures, offering new avenues for research and clinical application in understanding ocular diseases characterized by vascular and inflammatory components like BD.

Retinal function imager

Retinal functional imaging (RFI) represents a cutting-edge technology that promises to advance our understanding of capillary microvasculature by precisely measuring retinal blood flow velocity by detecting hemoglobin motion. This innovative approach allows for the visualization of microvascular dynamics and provides quantitative data on blood flow parameters within the retina. In BD, characterized by significant venous vascular involvement, RFI can accurately assess blood flow dynamics and potentially identify and quantify vascular abnormalities associated with BD, aiding in diagnosis and the ongoing management of the disease. The ability of RFI to detect subtle changes in retinal blood flow velocity and hemodynamics may provide insights into disease progression and response to treatment in BD patients [47]. Noninvasive ocular imaging is leading in front, including OCT and OCT-angiography, adaptive optics, and a few more commercially available modalities like retinal fiber imaging that can aid in preclinical diagnosis [38].

## Conclusions

Ocular BD plays an integral role in the diagnosis of systemic disease. However, we need to widen resources for early diagnosis with available technologies. Color fundus photography, fluorescein angiography (FFA), optical coherence tomography (OCT), and optical coherence tomography angiography (OCTA) collectively form the cornerstone of multimodal imaging for evaluating the posterior segment in patients with Behçet's uveitis. Each of these imaging modalities plays a crucial role in diagnosing, monitoring, and managing the ocular manifestations of this complex inflammatory disease. FFA traditionally serves as the gold standard for monitoring retinal vasculitis in Behçet's uveitis, providing detailed visualization of vascular leakage and abnormalities. However, the invasive nature of FFA limits its utility in frequent monitoring and in patients with contraindications to dye injection. This is where OCT and OCTA have emerged as indispensable tools due to their noninvasive capabilities and ability to provide high-resolution macula and retinal vasculature imaging. The advent of modern imaging devices has significantly enhanced our understanding of inflammatory lesions and structural changes in the posterior segment of the eye in Behçet's uveitis. These technologies aid in early diagnosis and guiding treatment decisions and assessing treatment response over time.
